# Determinants and Outcomes of the Therapeutic Alliance in Treating Justice-Involved Youth: A Systematic Review of Quantitative and Qualitative Research

**DOI:** 10.1007/s10567-022-00407-2

**Published:** 2022-08-16

**Authors:** Nina Papalia, Ashley Dunne, Natasha Maharaj, Erika Fortunato, Stefan Luebbers, James R. P. Ogloff

**Affiliations:** 1 Centre for Forensic Behavioural Science, Swinburne University of Technology and Victorian Institute of Forensic Mental Health, Level 1, 582 Heidelberg Road, Alphington, VIC 3078 Australia; 2 Psychology Department, John Jay College of Criminal Justice, New York City, NY USA; 3 Youth Forensic Specialist Service, Alfred Health, Moorabbin, VIC Australia

**Keywords:** Juvenile justice, Adolescence, Violence, Offending, Therapeutic alliance, Treatment

## Abstract

A large body of research supports the role of the therapeutic alliance in predicting positive change in psychotherapy. This systematic review examined determinants of alliance quality and its association with treatment outcomes in an under-served and under-researched population—justice-involved youth—with whom several challenges and contextual considerations arise that bear relevance to the alliance. The search strategy yielded 23 independent studies meeting eligibility criteria and describing diverse treatments: 14 quantitative records synthesized narratively and nine qualitative studies that underwent thematic analysis. A complex picture emerged, precluding firm conclusions about factors linked to enhanced alliances and the alliance–outcome relationship with justice-involved youth. Nevertheless, some promising findings were noted across quantitative studies, including potential treatment benefits related to alliance *growth* and creating positive alliances with caregivers. The review also highlighted the potential relevance of the young person’s relationships with peers and parents and their treatment readiness and expectations to alliance quality. Drawing on adolescent, caregiver, and therapist perspectives, the thematic synthesis of qualitative studies generated themes related to key elements of constructive alliances and their role in creating a foundation for initiating change. An integrated discussion is provided, highlighting practical implications and suggestions for addressing methodological limitations and substantive knowledge gaps.

## Introduction

Treatments that meet the needs of young people who engage in criminal behavior and reduce reoffending are critically important. This is underscored by the fact that a relatively small group of youth are responsible for a disproportionate number of juvenile offenses, including violent offenses, many of whom recidivate and progress to the adult justice system (Barrett & Katsiyannis, 2016; Garrido & Morales, 2007; Mulder et al., 2019; Sutherland & Millsteed, 2016). Moreover, alongside their offending-related treatment needs, justice-involved youth often present with other clinical conditions, including mental health and substance use disorders (Beaudry et al., 2021; Meurk et al., 2019). Left untreated, these problems can undermine a young person’s responsivity to interventions targeting criminal behavior. Supported by a robust research literature, the Risk-Need-Responsivity (RNR) model identifies the types of treatments that, all else being equal, most likely lead to reduced offending in general, and youth offending specifically (Bonta & Andrews, 2017): namely, treatments that match the young person’s empirically assessed risk level (i.e., more intense and extensive services are afforded to higher risk cases), target dynamic risk factors known to be associated with reoffending, adopt cognitive behavioral principles, and take an individualized, tailored approach that considers relevant responsivity issues (e.g., adapted to learning abilities, motivation level, trauma histories, culture, colonization and social marginalization experiences). Yet, a sizeable portion of justice-involved youth drop out of treatment, and treatment effects for those retained remain modest (Olver et al., 2011; Pappas & Dent, 2021). These treatment failures underscore the importance of knowing how service providers should deliver evidence-based treatments to maximize benefits to youth, their families, and the community. One therapeutic process variable, the therapeutic alliance, has received little attention in the juvenile justice realm compared to conventional psychotherapy and is the focus of this review.

The therapeutic alliance denotes some of the most important elements of the relationship between a client and therapist. Within the adult psychotherapy literature, the alliance is commonly conceptualized as comprising three interrelated components: (1) mutual agreement and understanding regarding the *goals* of therapy; (2) clear definition and negotiation of the *tasks* necessary to achieve these goals; and (3) the development of an affective *bond* or mutual trust between the parties (Bordin, 1979). Prior reviews consistently link strong therapeutic alliances to positive treatment outcomes across various treatment modalities (e.g., Baier et al., 2020; Fluckiger et al., 2018; Horvath & Bedi, 2002).

Alliance research in child and adolescent populations has progressed more slowly than the adult field. To date, two leading youth alliance conceptualizations have guided much of the research and development of measures to assess youth alliance (Karver et al., 2018). Drawing from psychodynamic perspectives (Freud, 1946; Meeks, 1971), the first conceptualization proffered by Shirk and Saiz (1992) emphasizes the alliance as a collaborative bond (captured via two dimensions of bond and task collaboration) between the youth and therapist. The second major conceptualization, which derives from Bordin’s (1979) model, emphasizes the youth alliance as a contractual bond comprising three interrelated dimensions of goals, tasks, and bond (DiGiuseppe et al., 1996). Although both perspectives propose multidimensional structures, findings have been mixed about whether these dimensions are empirically replicated in youth samples, with some work supporting a unidimensional structure (Faw et al., 2005; Ormhaug et al., 2015; Roest et al., 2016; Shelef & Diamond, 2008). This highlights that the core features of the alliance may be less differentiated for young people than adults (Shirk et al., 2011). Irrespective of the underlying structure, meta-analyses of child and adolescent clinical populations demonstrate small-to-modest associations between the alliance and treatment outcomes (Karver et al., 2018; McLeod, 2011; Shirk et al., 2011; Welmers-van de Poll et al., 2018).

Several challenges and considerations are relevant to therapeutic work with offending/justice-involved youth that might impact alliance formation and its relation to outcomes. Some are shared with young people in general (e.g., underdeveloped cognitive capacities, involvement of other family members, desire for increased autonomy from adults; DiGiuseppe et al., 1996; Karver et al., 2018; Shirk et al., 2011; Zack et al., 2007), while others are unique to or magnified by the justice context. For example, offending and justice-involved youth often possess shallow or only external treatment motivation, significant emotion regulation difficulties, high levels of hostility, severe conduct problems, and deficits in social-cognitive skills (Docherty et al., 2021; Kapoor et al., 2018; Tarolla et al., 2002). In addition, rates of child maltreatment, trauma, family disadvantage, disability, and mental illness are all disproportionately high in this population (Baidawi & Piquero, 2021; Beaudry et al., 2021; Fox et al., 2015; Papalia et al., 2022). Such characteristics may represent barriers to forming and maintaining productive therapeutic relationships. Similarly, young people from racially, culturally, and linguistically diverse backgrounds are over-represented in justice systems internationally. The unique contexts and stressors experienced by many of these youth and their families may be consequential for the therapeutic alliance and for clinicians’ efforts to develop trust and (cultural) safety when working cross-culturally.

Although client-therapist collaboration in setting the goals and tasks of treatment is critical to developing a strong alliance, in the case of offending behavior treatment programs, the goals and tasks of therapy are typically pre-determined with community safety considerations at the fore (Kozar & Day, 2012). Relatedly, while some justice-involved youth actively seek help, many do not self-refer to programs targeting offending, substance use, or mental health. Rather, they may be mandated, coerced, or pressured to receive treatment (Hachtel et al., 2019; Parhar et al., 2008; Smallbone et al., 2009). Clinicians working with youth in justice contexts may need, therefore, to balance dual roles: on the one hand being an agent of change, encouraging the young person to develop a trusting and self-disclosing relationship with them, but on the other hand being a figure of control, where they may disclose risk-relevant information to third parties about the young person (Kozar & Day, 2012; Skeem et al., 2007; Ward et al., 2015). Even if a therapist does not hold a dual role, the young person may view them as part of a coercive system, undermining the alliance.

Another complicating factor is that treatment approaches for justice-involved youth often include group interventions (or settings/environments) and family treatments (Kozar & Day, 2012; Lipsey, 2006; Pappas & Dent, 2021). While the therapeutic alliance–outcome relationship has been supported in group and family-involved therapies, some evidence suggests that the alliance may interact with other group (e.g., cohesion and climate) and family processes (e.g., parent alliance) in a complex interplay that influences treatment outcomes, both positively and negatively (Alldredge et al., 2021; van der Helm et al., 2009; Welmers-van de Poll et al., 2018). Similarly, it is not unusual for multiple therapeutic (or support) staff to be involved in caring for justice-involved youth, each relationship of varying importance and quality. It is plausible that how the alliance develops and whether it is linked to change is influenced by the complex array of relationships the young person (and their family) forms with their care team.

Recognizing the complexities of therapy in criminal justice contexts, Ross et al. (2008) revised Bordin’s (1979) alliance concept to provide a more elaborate theory of how the alliance develops and is maintained in therapy with offending adults. In the revised model, therapist characteristics (e.g., personality, professional/interpersonal skills, biases, expectations of the client), client variables (e.g., irritability, callousness, attachment insecurity, treatment readiness), and their interaction (e.g., (dis)similarity in values, matched cultural backgrounds), are theorized to influence therapist and client cognitive processes and emotional reactions to each other and the therapy process. These processes and responses manifest as in-therapy behaviors that directly feed into the bond, goals, and tasks dimensions of the alliance, and which are also affected by the broader context in which therapy is delivered (Ross et al., 2008). Contextual factors might include, for example, justice system/organizational policies, availability of pleasant and safe therapy spaces, level of therapist supervision and reflective practice, whether treatment is delivered in a group setting, and so on. Later, Orsi et al. (2010) extended Ross et al.’s model to elucidate additional factors that might influence the working alliance^1^ for adolescents in formal “authoritarian” settings (e.g., child welfare/residential care settings, substance use treatment, probation). Key additions included the potential role of youth developmental stage and their social networks (e.g., families, peers, school) in how the working alliance forms.

Despite the clinical coherence of the abovementioned models, to our knowledge, there are no published systematic reviews of empirical work concerning the determinants of the therapeutic alliance with offending/justice-involved youth. Further, despite the robust association between the alliance and treatment change in conventional psychotherapy, very little is known about the alliance’s role in creating change in justice-involved youth. A review of research on the alliance in adult violent offending behavior programs concluded that while there are clear theoretical and practice grounds for developing a strong alliance, there is insufficient data to determine whether it directly or indirectly impacts treatment outcomes in this population (Kozar & Day, 2012). It is unknown whether conclusions from the broader youth therapy literature or the adult correctional treatment literature about the nature and value of the alliance can be applied to treatments with justice-involved youth.

To address these knowledge gaps, we conducted a systematic review of studies investigating the therapeutic alliance in treating offending/justice-involved youth. Our primary objectives were to synthesize quantitative research on (1) the determinants of a positive therapeutic alliance; and (2) the relationship between alliance quality and treatment outcome. In addition, we sought to synthesize qualitative research on the perspectives of young people, their caregivers, and treatment providers about the nature and role of the alliance in this context. This qualitative strand was more exploratory and aimed to include, for example, views about the features of a positive alliance, factors that facilitate or hinder alliance formation, and the relevance and value of the alliance in generating therapeutic change.

## Methods

This review was prepared using the Preferred Reporting Items for Systematic reviews and Meta-Analyses guidelines (PRISMA; Page et al., 2021) and the Enhancing Transparency in Reporting the Synthesis of Qualitative Research statement (ENTREQ; Tong et al., 2012). Although the review was not registered, review methods were determined a priori and written into a protocol, available from the authors.

### Eligibility Criteria

#### Population

The target population comprised adolescents (i.e., aged 10–17 years, or present in a juvenile facility or service) with justice involvement and/or a history of criminal behavior (i.e., behavior that would be grounds for arrest, irrespective of whether criminal sanctions were imposed). In most instances, justice involvement and offending were derived from official sources (e.g., incarceration, convictions), with a minority of studies using other sources (e.g., self- or informant-reported delinquency and violence). Where studies used mixed samples (e.g., justice-involved and child welfare-involved youth), we required that ≥ 75% comprised justice-involved/offending youth or that analyses were reported separately for this subgroup. The following studies were excluded: samples with a mean age below 10 years; samples defined by general or composite constructs like externalizing/disruptive behavior, aggression, and substance misuse, that did not clearly meet population criteria; and samples with primarily developmental status offenses or “soft delinquency”, that is, non-criminal offenses that are law violations due to minor status (e.g., truancy, running away from home, violating curfew).

#### Study Designs/Settings and Evidence Sources

All records were required to describe original empirical research. Studies that used quantitative or qualitative methods were eligible for inclusion, but we excluded case studies. All types of study/treatment settings were eligible. Both peer-reviewed research studies and dissertations were eligible for inclusion, providing they were in full-text form and written in English. No limits were placed on publication year.

#### Therapeutic Alliance

Quantitative studies must have included a measure of the therapeutic alliance (or treatment alliance, helping alliance, working alliance) or one or more of its key components (e.g., mutual affective bond, collaborative goals, and/or task orientation). Furthermore, the associations relevant to the review objectives had to be tested statistically. For qualitative studies, eligible studies were required to either directly inquire about the alliance or identify the alliance (or one or more of its components) as a resultant theme from participant responses. We did not place restrictions on the method or timing of alliance assessment.

Only studies where the target therapeutic alliance occurred in the context of therapeutic/clinical treatment were eligible for inclusion, i.e., we excluded non-therapeutic contexts (e.g., mentoring programs, employment/educational programs). Although we did not place limits on target treatment problems (e.g., offending behavior, mental health, substance use), there must have been a broadly stated aim to address problematic/antisocial behaviors, reduce psychological distress, or increase prosocial and adaptive functioning. Eligible target alliances were between the practitioner(s) providing the treatment and the young person or parent/caregiver receiving the treatment (i.e., youth–therapist alliance or parent–therapist alliance). Correctional relationships (e.g., with correctional officers, youth justice case managers) that served a surveillance function rather than, or alongside, a rehabilitative role were not eligible for inclusion.

#### Alliance Determinants and Outcomes

For studies with analyses relevant to our first review objective (factors associated with alliance quality), we took an inclusive approach and considered all potential factors reported in studies. Only factors measured prior to or concurrent with the alliance were eligible. Studies relevant to our second objective (alliance–outcome relationship) were required to measure and report at least one outcome variable. Primarily, we were interested in ‘treatment’ outcomes; that is, those factors directly targeted by treatment or might otherwise be reasonably expected to change because of treatment. However, given the anticipated limited literature concerning the alliance in justice-involved youth, along with the challenges associated with effectively engaging this group in treatment, we also included studies examining ‘process’ outcomes (e.g., dosage, treatment completion, therapist adherence). We did not require temporal separation between alliance and outcome measures; both prospective and concurrent associations were eligible.

### Literature Search Strategy

To locate eligible studies, we searched several databases from inception to March 3rd, 2021: PsycINFO; MEDLINE; Criminal Justice Abstracts with Full Text (EBSCOhost); ProQuest (Social Science Premium Collection); and CINCH Australian Criminology Database. For each database, we used the following search terms: “offen*” OR “violen*” OR “aggress*” OR “delinquen*” OR “criminal behavio?r*” OR “justice-involved” AND “therapeutic alliance” OR “working alliance” OR “helping alliance” OR “therapeutic relationship” AND “youth*” OR “juvenile*” OR “adolescen*” OR “young people*” OR “young offender*” OR “teenager*”. After the initial search, the references of previous systematic reviews on the youth alliance–outcome relationship were examined as well as references from potentially suitable papers. A forward citation search (in Scopus) was conducted of three existing reviews and two oft-cited studies relevant to the alliance in forensic and justice settings (Florsheim et al., 2000; Holmqvist et al., 2007; Kozar & Day, 2012; Orsi et al., 2010; Ross et al., 2008). The search strategy was updated on February 4th, 2022, to include any eligible studies published since the original search.

### Study Selection

Study selection was undertaken by two authors (N.P. and A.D.) using *Covidence* systematic review software. After removing duplicate records, both reviewers independently screened 93% of titles and abstracts to remove irrelevant documents, with 90% agreement (the remaining 7% of records were identified via the updated search and screened by N.P. only). Disagreements were resolved through discussion, with decisions favoring an inclusive approach at this review stage. Next, full-text articles of screened-in records were obtained and assessed against inclusion criteria. A sample of 24 full-text records (21%) was independently reviewed by both authors, with 92% agreement. Decisions on the remaining full-text records were made by one author (N.P.). Where there was insufficient information to determine study eligibility, attempts were made to contact authors; where unsuccessful, ambiguous records were excluded.

### Data Extraction

Two data extraction forms were developed (one for quantitative and qualitative studies), including all variables for which data were sought from primary studies. To establish the forms, content areas of interest were identified and items were developed to assess these areas. Next, pairs of reviewers (N.P., A.D., N.M.) independently piloted the coding forms using a small subsample of included records (*n* = 3), with 88% agreement. All items were reviewed, discrepancies were resolved, and poor coding items were revised to improve accuracy and agreement. The final coding forms examined several content areas: author and study descriptors (e.g., year, location, design); sample descriptors (e.g., youth age, gender, race/ethnicity); treatment descriptors (e.g., target problem, treatment model, setting, duration); alliance descriptors (e.g., alliance dimension(s), target relationship, rating source, measure(s), timing); alliance determinants and outcomes (e.g., variables/outcomes assessed, rating source, measure(s), timing); and a summary of key analyses/findings and authors’ conclusions. Finally, authors N.P. and N.M. extracted data for all remaining quantitative and qualitative studies, respectively.

### Quality Assessment

The Mixed Methods Appraisal Tool (MMAT) Version 18 (Hong et al., 2018) was used to appraise included studies critically. The tool consists of separate sections for quantitative and qualitative methodologies. Quantitative analyses were assessed across five methodological criteria: sample representativeness; measurement of the independent and dependent variables; completeness of outcome data; confounding influences; and, where applicable (longitudinal studies only), changes in exposure (independent variable) status over the study period and potential co-exposures. Each criterion was rated as ‘Yes’ (indicating sufficient methodological quality such that plausible issues would be unlikely to alter results seriously), ‘No’ (indicating insufficient methodological quality such that plausible problems seriously weaken confidence in the results), or ‘Cannot Tell’ (the study did not report appropriate information to answer ‘Yes’ or ‘No’ or reports unclear information). The appraisal of qualitative studies involved assessing the congruence of the aims of each study to five domains, namely, qualitative methodology, methods, analysis, findings, and reporting.

In accordance with MMAT recommendations, we developed a list of specific indicators for each domain and applied these uniformly across all quantitative and qualitative studies, respectively. Quality criteria were rated at the review objective level. Two authors independently rated quality for four quantitative studies (N.P. and A.D., with 80% agreement) and two qualitative studies (N.M. and N.P., with 100% agreement). Disagreements were resolved through discussion, and rating indicators were clarified and expanded where necessary to improve consistency. The remaining studies were appraised by one author (N.P. or N.M.).

### Results Synthesis

Regarding quantitative research, we anticipated heterogeneity in the types of treatment programs under consideration and alliance determinants and outcomes. As such, we planned to narratively synthesize findings rather than conduct meta-analyses. Results were synthesized first according to review objective and then by key variable domains. Qualitative research was synthesized using thematic analysis. This was initiated with line-by-line coding of results/findings text of primary studies. Free codes were then organized into descriptive themes. Interpretation resulted in the development of overarching analytical themes across studies that went beyond the interpretation in the primary analyses. Further organization resulted in a synthesis of findings represented by themes and sub-themes, illustrated in tabular form, and elaborated on in a descriptive narrative.

## Results

### Study Selection and Characteristics

**Fig. 1:**
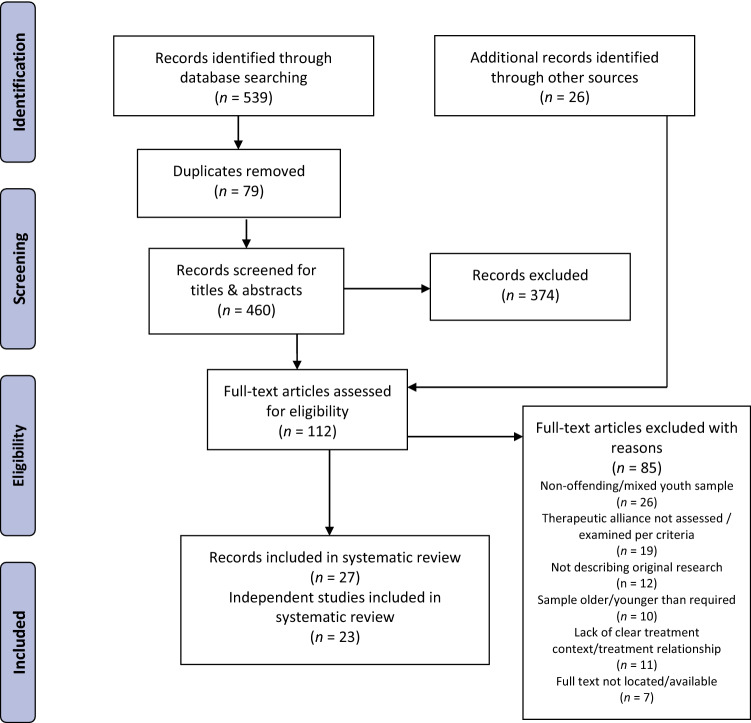
PRISMA diagram depicting the flow of studies from identification to inclusion

Figure 1 depicts the study selection process. There were 112 records that underwent full-text appraisal. Of these, 27 records (comprising 23 independent studies) satisfied eligibility criteria, comprising 14 quantitative studies (from 17 records) and nine qualitative studies (from 10 records). Shown in Table 1, records were published between 2000 and 2020, comprising mostly journal articles (*n* = 19; 70.4%) and samples from the US (*n* = 21; 77.8%). The majority (*n* = 15; 88.2%) of quantitative records used prospective-longitudinal designs, whereas a grounded theory approach was most frequently adopted in qualitative records (*n* = 5; 50%). The average of mean ages across youth samples was 15.9 years. Most youths were male and from minority racial/ethnic backgrounds. Treatments were diverse but most frequently addressed offending/externalizing behavior as the primary target (*n* = 18; 78% of independent treatment samples) and were delivered in community and home-based settings (*n* = 14; 61%)**.** Tables 2 and 3 summarize characteristics related to the therapeutic alliance assessment from quantitative and qualitative studies, respectively.

**Table 1:** Descriptive data for the 23 independent studies (27 records) included in the review

Study, country	Description of the youth sample	*M* youth age (years)	Male youth (%)	Youth race/ethnicity (%/*n*)	Design/approach	Primary target problem	Tx type(s)	Tx setting(s)	Tx length
Quantitative studies				% Reported					
Florsheim et al., 2000, US	76 delinquent youth in community-based treatment	15.6	100	37 Ethnic minorities^e^ 63 Caucasian	Prospective-longitudinal	Offending/externalizing	ND	Residential	NR
**Dauber,** 2004, **US**^**a**^; **Alkalay,** 2006, **US**^**a,b**^	**113 justice-involved substance-abusing adolescents drawn from larger RCT**	**15.3**	**79**	**68 African American** **13 Hispanic** **19 Caucasian**	**Prospective-longitudinal**	**Substance use**	**MDFT, CBT**	**Community**	**16–24 weeks**
**Hogue et al.,** 2006, **US**	**100 justice-involved substance-abusing adolescents drawn from larger RCT**	**15.5**	**81**	**68 African American** **12 Hispanic** **20 Caucasian**	**Prospective-longitudinal**	**Substance use**	**MDFT, CBT**	**Community**	**16–24 weeks**
Holmqvist et al., 2007, SE	59 youth in juvenile offender institutions	17.2	100	NR	Prospective-longitudinal	Offending/externalizing	ND	Custodial	*M* = 1 year, 2 months
**Shelef et al.,** 2005, **US**	**65 substance-abusing adolescents drawn from larger RCT; 71% had histories of violence**	**16**	**85**	**47 African American** **47 White**	**Prospective-longitudinal**	**Substance use**	**MDFT**	**Community**	**12 weeks**
**Diamond et al.,** 2006, **US**	**356 substance-abusing adolescents drawn from larger RCT; 66% had histories of violence**	**15.7**	**81**	**31 African American 61 Caucasian non-Hispanic** **8 Other**	**Prospective-longitudinal**	**Substance use**	**MDFT, MET + CBT,** **FSN, ACRA**	**Community**	**5–12 weeks**
Handwerk et al., 2008, US	71 delinquent youth from an out-of-home family style residential facility referred to onsite psychotherapy clinic	15.9	52	NR	Prospective-longitudinal	Offending/externalizing	Psychotherapy	Residential	*M* = 13 sessions
Savicki, 2008, US^a^	114 incarcerated mostly violent youth	18.4	84	31 Black 35 Latino 29 White 4 Multiracial 1 Native American	Prospective-longitudinal	Offending/externalizing	ND	Custodial	NR
Ryan et al., 2013, US	185 adolescents with crimes against another person, property offenses, substance abuse, or other externalizing behavior problems	15.4	65	28 Hispanic/Latino 20 African American 48 Caucasian 4 Other	Prospective-longitudinal	Offending/externalizing	MST	Home	NR
Simpson et al., 2013, US	58 adjudicated delinquents identified to be in need of mental health services; 55% with violent offenses	16.8	100	67 African American 28 Caucasian 2 Hispanic 2 Native American	Cross-sectional	Mental health, Substance use	CBT/MI, CYT	Custodial	*M* = 281 days^c^
The MVPP, 2014, US	334 high-risk youth (6th graders) with histories of violence drawn from larger RCT	NR	65	65 African American or Black 16 Latino/Hispanic	Prospective-longitudinal	Offending/externalizing	MVPP	School	15 weeks
Bovard-Johns et al., 2015, US	332 juveniles with adjudicated sexual offenses in residential treatment	16.7	100	26 African American 8 Native American 7 Hispanic 46 Caucasian 13 Missing	Cross-sectional	Offending/externalizing	SOP	Custodial	NR
Lange et al., 2017, NL	1970 adolescents with severe externalizing behavioral problems	15	69	24 Nonwestern origin	Prospective-longitudinal	Offending/externalizing	MST	Home	Up to 5 months
Mattos et al., 2017, US	59 delinquent adolescents drawn from larger randomized naturalistic trial	15.3	49	64 Hispanic 15 African American 7 Multiracial	Prospective-longitudinal	Offending/externalizing, substance use	Psychotherapy	Community	*M* = 3–4 months
Glebova et al., 2018, US	164 youth with antisocial behavior problems	15	65	26 Latina/o 20 Black 51 White 3 Other^d^	Prospective-longitudinal	Offending/externalizing	MST	Home	3–5 months
Cosgrove, 2020, US^a^	Up to 577 community-dwelling court/justice-involved youth	15.7	77	55–58 Black non-Hispanic 16–17 Hispanic /Latino or other 27–28 White non-Hispanic^f^	Prospective-longitudinal	Offending/externalizing	FFT	Home	3–5 months
Qualitative studies				*n* Reported					
Church, 2008, US^a^	Nine female adolescents court-mandated to receive therapy; two-thirds with violent offenses	NR	0	2 Caucasian/Latina, 1 African American, 1 Mexican, 1 Caucasian Portuguese, 1 Asian American, 1 African American/Latina, 1 Latina, 1 Caucasian	Qualitative description	Offending/externalizing	Psychotherapy	Community	*M* = 4–5 months
Ryals, 2011, US	Eight adolescents on probation	NR	75	3 African American, 4 Caucasian, 1 Hispanic	Phenomenology	Offending/externalizing	Psychotherapy	Community	At least 8 sessions
Tighe et al., 2012, UK	16 community-dwelling justice-involved youth	15.3	81	9 Black, 8 White, 3 Mixed, 1 Asian^g^	Qualitative description	Offending/externalizing	MST	Home	3–6 months
Schiller, 2013, CA^a^	Four adolescents adjudicated for sexual offenses	NR	100	NR	Phenomenology	Offending/externalizing	SOP	Residential	At least 6 weeks
Brown et al., 2014, US	19 previously detained youth referred for mental health treatment	16 (F) 15.5 (M)	63	10 Black, 1 Hispanic, 8 White	Grounded theory	Mental health	ND	Community	NR
Yoder, 2013^a^; Yoder & Ruch, 2016, US^b^	Adolescents adjudicated for sexual offenses	NR	NR	NR	Grounded theory	Offending/externalizing	FT	Mixed	NR
Paradisopoulos et al., 2015, UK	Eight adolescents with serious antisocial and conduct problems	NR	38	1 Black Caribbean, 2 Mixed, 5 White British	Grounded theory	Offending/externalizing	MST	Community	3–5 months
Kaur et al., 2017, UK	10 adolescents with serious antisocial and conduct problems	NR	40	1 Black British Caribbean, 11 White British^d^	Grounded theory	Offending/externalizing	MST	Community	3–5 months
Sanches-Vasquez, 2016, US^a^	10 justice- and gang-involved, Mexican–American youth on probation	16.5	100	10 Mexican–American	Phenomenology	Offending/externalizing	Psychotherapy	Community	NR

Adjacent bold rows reflect studies with overlapping samples. % values are rounded to nearest whole number

*M* mean, *Tx* treatment, *US* United States of America, *SE* Sweden, *UK* United Kingdom, *CA* Canada, *MVPP* multisite violence prevention project group, *NL* Netherlands, *ND* not defined, ill-defined or highly mixed treatments, *NR* not reported, *RCT* randomized controlled trial, *MDFT* multidimensional family therapy, *CBT* cognitive behavioral therapy, *MET* + *CBT* motivational enhancement therapy plus CBT, *FSN* family support network, *ACRA* adolescent community reinforcement approach, *MST* multisystemic therapy, *MI* motivational interviewing, *CYT* cannabis youth treatment, *SOP* sexual offending programming, *FFT* functional family therapy, *F* females, *M* males, *FT* family treatment

^a^Indicates dissertation

^b^Records in the same row use the same study sample and are thus reported on a single line

^c^Length of time in the facility was used in this study as a proxy for length of time in treatment

^d^Studies report race/ethnicity of the youths’ caregivers

^e^Includes Latino, African American, Native American, Asian American, and Pacific Islander

^f^Range of values reported due to different samples used in main analyses

^g^Includes five youth who did not participate in an interview

**Table 2:** Therapeutic alliance descriptive information for the 14 independent quantitative studies (17 records) included in the review

Study	Therapeutic alliance (TA) measure	TA rel.	TA Ax	TA timing^a^	RO1	RO2	RO1 domains	RO2 domains
Florsheim et al., 2000	Working Alliance Inventory	YT	Y, T	Early, Late Change	√	√	Antisocial behavior; family & peer factors	Antisocial outcomes; mental health; Tx completion
**Dauber,** 2004^**b**^; **Alkalay,** 2006^**b,c**^	**Revised Vanderbilt Therapeutic Alliance Scale**	**YT, PT**	**O**	**Early, averaged**	**√**	**√**	**Antisocial behavior; mental health; socio-demographics; family & peer factors; program factors**	**Antisocial outcomes; mental health**
**Hogue et al.,** 2006	**Revised Vanderbilt Therapeutic Alliance Scale**	**YT, PT**	**O**	**Early, change**	**√**	**√**	**Socio-demographics; family & peer factors; program factors**	**Antisocial outcomes; mental health; Tx attendance**
Holmqvist et al., 2007	Helping Alliance Questionnaire and Interviews	YT	Y, T	Averaged	√	√	Tx perceptions/expectations	Antisocial outcomes
**Shelef et al.,** 2005	**Working Alliance Inventory—Short Form**	**YT, PT**	**Y, O**	**Early**	**√**	**√**	**Family & peer factors**	**Antisocial outcomes; Tx attendance**
**Diamond et al.,** 2006	**Working Alliance Inventory—Short Form**	**YT**	**Y, T**	**Early**	**×**	**√**	**–**	**Antisocial outcomes; Tx attendance**
Handwerk et al., 2008	Working Relationship Scale	YT	Y, T	Averaged	×	√	–	Antisocial outcomes; Mental health
Savicki, 2008^b^	Working Alliance Inventory—Short Form	YT	Y	NR	√	√	Tx perceptions/expectations	Antisocial outcomes; Tx progress
Ryan et al., 2013	Emotional Bonding Subscale of the Working Alliance Inventory	YT, PT	Y, P	Mid, late, averaged	√	×	Antisocial behavior; socio-demographics; family & peer factors; therapist factors	–
Simpson et al., 2013	Working Alliance Inventory	YT	Y, T	281 days on average post-intake	√	√	Antisocial behavior; personality; Socio-demographics	Antisocial outcomes; Tx attendance
The MVPP, 2014	Questionnaires measuring trust/positive feelings and helpfulness of an alliance	YT, PT	Y, T, P	Early, change	√	√	Antisocial behavior; socio-demographics; family & peer factors; Tx perceptions/expectations	Parental functioning; Tx attendance; Fidelity
Bovard-Johns et al., 2015	Helping Alliance Questionnaire–II	YT	Y	1.2 years on average post-intake	√	×	Mental health; family & peer factors	–
Lange et al., 2017	Therapist Adherence Measure Revised	YT, PT	P	Early, Mid, Late	√	√	Therapist factors	Fidelity
Mattos et al., 2017	Therapeutic Alliance Scale for Adolescents	YT	Y	3 months post-intake	√	√	Antisocial behavior; mental health and personality; socio-demographics	Antisocial outcomes; Tx attendance
Glebova et al., 2018	Emotional Bonding Subscale of the Working Alliance Inventory	YT, PT	Y, T, P	Mid, late	√	√	Family & peer factors	Antisocial outcomes; parental functioning; Tx progress
Cosgrove, 2020^b^	Family Self Report	YT, PT	Y, P	Early, mid, late, change	√	√	Antisocial behavior; socio-demographics; therapist factors	Antisocial outcomes; Tx completion; Fidelity

Adjacent bold rows reflect studies with overlapping samples

*Rel*. relationship, *Ax* assessor, *Tx* treatment, *RO1* study reports analyses relevant to review objective one (determinants of alliance quality), *RO2* study reports analyses relevant to review objective two (alliance–outcome relationship), *YT* youth and treatment provider alliance, *PT* parent/caregiver and treatment provider alliance, *Y* youth, *T* treatment provider, *O* observer, *P* parent/caregiver, *NR* not reported

^a^Indicates timing of the TA assessment relative to treatment. Can include early, mid, late (incl. end of) treatment, as well as averaged alliance scores or change across treatment

^b^Indicates dissertation

^c^Records in the same row use the same study sample and are thus reported on a single line

**Table 3:** Therapeutic alliance descriptive information for the nine independent qualitative studies (10 records) in the review and themes derived from synthesis

Themes and sub-themes	Study	Therapeutic alliance (TA) directly assessed	Target TA relationship	TA perspective	Themes	Sub-themes
i. Setting Treatment Goals, Tasks/Progress and Future Goals 1.Self-determination 2.Drawing on personal strengths 3.Collaboration 4.Active therapeutic engagement ii. Developing a Bond with the Therapist 5.Individualized understanding and family engagement 6.A trauma sensitive approach 7.Therapists’ traits/skills iii. Improving Capacity for Positive Relationships 8.Improved family involvement, resilience, and functioning 9.Positive peer influences 10.Improved engagement in education 11.Youth reflecting on behavior and becoming aspirational	Brown et al., 2014	Yes	Youth-therapist	Youth	i	1
					ii	6, 7
	Church, 2008^a^	Yes	Youth-therapist	Therapist	i	1, 3, 4
					ii	6
					iii	8
	Kaur et al., 2017	No	Parent-therapist	Parent/Caregiver	i	1, 2
					ii	
					iii	8
	Paradisopoulos et al., 2015	No	Youth-therapist	Youth	i	1, 3, 4
					ii	6, 7
					iii	8, 9, 11
	Ryals, 2011	Yes	Youth-therapist	Youth	ii	7
					iii	9, 10, 11
	Sanchez-Vasquez, 2016^a^	Yes	Youth-therapist	Youth	i	1, 2
					ii	5, 7
					iii	9, 11
	Schiller, 2013^a^	Yes	Youth-therapist	Youth	i	1, 3, 4
					ii	5
	Tighe et al., 2012	No	Youth-therapist Parent-therapist	Youth Parent/Caregiver	i	1, 3, 4
					ii	
					iii	8, 9, 10, 11
	Yoder, 2013^a^; Yoder & Ruch, 2016^b^	No	Youth-therapist Parent-therapist	Therapist	i	2
					ii	5
					iii	8

^a^Indicates dissertation

^b^Records in the same row use the same study sample and are thus reported on a single line

### Quality Assessment Results

#### Quantitative Studies

Only four study samples (out of 16 samples; 25%) demonstrated adequate representativeness of the target population. Problems in this domain were often the result of convenience, selective, or poorly defined sampling methods and participant response rates lower than 70%. Most studies used adequate measures to assess key independent and dependent variables (87% and 80% of records relevant to review objectives one and two, respectively) as judged primarily by reliability and validity indicators. Positively, 73% and 80% of records relevant to review objectives one and two, respectively, included non-shared measurement sources in analyses of key independent and dependent variables.

Up to 40% of records had problems related to missing outcome data, where fewer than 70% of participants contributed to key outcome measures. Inadequate information on missingness was a problem in a significant portion of the remaining records. In assessing confounding influences, we evaluated whether authors considered the issue of confounding in the design/analysis and whether they adequately accounted for these through appropriate methods. For records relevant to review objective one, 20% were judged to be adequate in this domain, whereas 67% of review objective two records considered confounding influences.

Changes in exposure (independent variable) status over the study period and co-exposures were conceptualized as potential confounding influences that arose subsequent or concurrent to the independent variable. Plausible problems were evident in this domain for 33% and 73% of records relevant to review aims one and two, respectively. Issues were frequently related to inconsistency in the treatments received (e.g., focus, length, exposure to multiple interventions) and mechanisms to ensure treatment integrity/fidelity, and a lack of consideration of changes in the alliance over time or the influence of multiple alliances formed by the young person.

#### Qualitative Studies

All qualitative studies were deemed sufficiently well-conducted overall. The methodological approaches and data collection methods were appropriate to achieve the aims/objectives of all studies; all used semi-structured interviews. However, rigor could have been enhanced in some studies. For example, data saturation was not discussed in five (56%) studies, the recruitment strategy was not detailed in three (33%) studies, and in four (44%), the rationale for recruitment strategy was not clearly articulated.

Results were analyzed using some form of thematic analysis in all studies, which was appropriate. In addition, all studies provided an in-depth explanation of the analytical process, demonstrating that findings were adequately derived from the data and providing sufficient description with which to assess rigor. Further augmenting the quality of some studies was the added indicator of ‘reflexivity’ for this criterion, where researchers consider their role and bias in the analysis, which can strengthen the interpretation of the assumptions and findings made; four (44%) studies made no mention of this concept.

Interpretation of results was well substantiated by quotations of participants’ perspectives in all studies. Further enhancing the quality of all studies was a demonstration of rigor and trustworthiness of the interpretive process, with each study illustrating this adequately through various means, congruent with their methodological approach. This included data triangulation, other coders/analysts, other reviewers, respondent validation/member checks, and an audit trail.

Reporting of research demonstrating coherence between the different stages of the research process was well articulated by all studies. There were clear links between research objectives, methodology, methods, analysis, and interpretation of findings. Most studies discussed implications for clinical practice and suggestions for future research, providing an assessment of the utility of findings. However, this discussion was limited in two (22%) studies.

### Results Synthesis

Given the exploratory nature of the qualitative strand, we begin by presenting the themes emerging from qualitative research, drawing from adolescent, caregiver, and therapist perspectives. We then offer the more targeted synthesis of quantitative studies examining the determinants of a positive alliance and alliance–outcome relationship.

#### Qualitative Research

Of the nine qualitative studies, five directly investigated the alliance and four elicited this concept through study findings. The synthesis garnered three overarching themes. Two themes reflected perspectives on the nature and features of a positive alliance, namely, ‘setting treatment goals, tasks/progress, and future goals’ and ‘developing a bond with the therapist.’ The last theme, ‘improving capacity for positive relationships’, pointed to potential outcomes linked to positive alliances. All three themes elicited sub-themes, which further elucidated perspectives on the important facets of a positive alliance and its relevance to treatment outcomes (see Table 3). Following is a succinct summary of these themes and sub-themes:

##### Setting Treatment Goals, Tasks/Progress, and Future Goals

Eight studies elicited sub-themes around the first theme encompassing the importance of a shared sense of goals and tasks in treatment that define progress and planning for future goals.

###### *Self-determination*

Seven studies revealed that self-determination, or the ability to have choice and agency by being an active participant in generating goals, was influential in developing the alliance and achieving positive outcomes. For example, Church (2008) spoke of “shifting the power in the relationship to the client” (p. 49) to illustrate the importance of empowerment in the therapeutic process. A young person in one study spoke of feeling empowered by the therapeutic approach to choose a different future:

I’ve stayed in college. I was very surprised about that. I know my mum was surprised at that but I stayed in college for 2 years. I’ve definitely got my head on my shoulders. I know what I want to do now for a fact so that’s really good. (Sammi, 18) (Paradisopoulos et al., 2015, p. 484)

###### *Collaboration*

In several studies, collaboration with therapists on deciding goals and tasks of treatment appeared to enable young people and their families to see themselves as agents in the change process. For example, in Schiller (2013), young male participants were reported to want to work with their counselor in deciding how their treatment would progress and be equals in the therapeutic relationship. In addition, a parent from Tighe et al. (2012) commented on how collaboration with families gave them the sense that their involvement in treatment was important:

It was all working together. Everybody had their part to play. You know, you owned something, which was quite good, especially for young people, they need to feel part of something. (P28) (Tighe et al., 2012, p. 191)

###### *Drawing on Personal Strengths*

Drawing on adolescents’ strengths was a technique used by therapists in some studies to provide positive messages about youths’ individual qualities and ways these qualities could assist them in achieving their goals and making progress. This seemed to both contribute to, and be facilitated by, a positive therapeutic relationship. For example, in Sanchez-Vasquez (2016), a youth spoke of understanding the need to utilize their strengths to help meet their goals:

[…] like she said I have good skills and she would always tell me that I am a good kid. I just needed to make better choices. I liked to hear that. (Participant 2) (Sanchez-Vasquez, 2016, p. 53)

###### *Active Therapeutic Engagement*

In several studies, engagement in practical tasks and activities early in treatment benefited the alliance. Youth and families tended to reflect positively on their relationships with therapists and perceive treatment as useful when they saw benefits early (e.g., received practical supports, learned new skills). A female adolescent participant pointed out an effective strategy she was asked to employ:

It was just the things I was getting told to do like go upstairs, calm down, like write my feelings down and that. (Louise, 14) (Paradisopoulos et al., 2015, p. 484)

##### Developing a Bond with the Therapist

All studies elicited sub-themes relevant to developing an affective bond during treatment and its importance in creating a trusting, safe environment for building engagement and the alliance.

###### *Therapists’ Traits/Skills*

Four studies referred to therapists’ traits and skills as influencing the development of a therapeutic bond. Traits and skills such as empathy, honesty, and genuineness were critical to developing safe and trusting working relationships. Youth also valued therapists being direct with them while creating rapport through humor. An adolescent spoke of this ‘straight up’ yet casual approach:

Like me, I like a person that’s a 100% with you, they’re truthful to you, they’re always wanting to joke around a little bit and have fun while you’re actually doing something, but mainly they are for real with you. They don’t joke around like they don’t beat around the bush or anything. They just tell you straight up. (17-year-old White male) (Brown et al., 2014, p. 200)

###### *Individualized Understanding and Family Engagement*

Some studies identified the importance of a flexible and accessible approach that adapted to the individual needs of young people and their families in developing the therapeutic bond. For example, Yoder and Ruch (2016, p. 199) commented that with therapists “giving parents and caregivers personalized information pertaining to youth’s behaviour… unique patterns of their child, or problem areas that could increase the likelihood for relapse,” the therapeutic relationship is strengthened, as are families’ understanding and knowledge of how to manage high-risk situations and prevent further offending. A treatment provider commented on this responsiveness to individuals’ needs:

Educating them [the families] on the cycle, triggers, and getting information from them [the families] about their own relations or what they see happening when the child is acting out. (Terri—treatment provider) (Yoder & Ruch, 2016, p. 199)

###### *A Trauma Sensitive Approach*

Youth having a voice, feeling understood and accepted, and safe in therapy were identified in some studies as important to developing the bond. For example, the perspectives of therapists of female adolescents in Church (2008) demonstrated the importance of presenting themselves in a non-blaming way so that youth feel heard and valued. An adolescent from Paradisopoulos et al. (2015) also spoke of the need to have a voice and to feel understood and safe in therapy:

In the beginning ... I found it awkward anyway to talk. But she made me feel comfortable as if I can say stuff and that she listened and not only gave – it wasn’t like a one-sided thing, she gave her point of view and she saw it from other people’s point of view and she saw it from mine which made me feel more comfortable. (Sammi, 18) (Paradisopoulos et al., 2015, p. 482)

##### Improving Capacity for Positive Relationships

The third theme centered on treatment outcomes, particularly the capacity for improved family relationships facilitated by the relational and technical aspects of treatment. Seven studies elicited sub-themes in this domain.

###### *Improved Family Involvement, Resilience, and Functioning*

Although treatment outcomes were often mixed (Tighe et al., 2012), five studies suggested a strong therapeutic relationship and empirically supported skills-based interventions were essential to achieve positive outcomes, such as improved family involvement, resilience, and functioning. For example, a service provider from Yoder and Ruch (2016) spoke of how they unite families by working on skills required for positive relationships:

You know, [I demonstrate] how to respectfully talk to each other and listen and not feel like you [the families] have to be defensive. You have to sort of model for them [the families] and help reframe so they know how to say it. I spend more time with clients teaching them how to say it. We will…like…practice. (Service Provider Patty) (Yoder & Ruch, 2016, p. 2000)

###### *Positive Peer Influences, Improved Engagement in Education, and Youth Reflecting on Behavior and Becoming Aspirational*

Some studies highlighted the relevance of the alliance in improving relationships outside of the treatment relationship. For example, a strong alliance with treating staff fostered re-inclusion in education. Furthermore, it helped young people imagine a more hopeful future, and some youth spent more time with prosocial peers, as articulated by one adolescent:

Just the way I see things has changed . . . like my friends, I started to realize they’re not clever . . . like before I’d do what they’d say. I hang around with more sensible people than before . . . sometimes I’m silly, but not enough to get myself involved with the police. (YP54) (Tighe et al., 2012, p. 193)

#### Quantitative Research

##### Determinants of Alliance Quality

Overall, 15 records provided quantitative data on factors associated with alliance quality, synthesized below.

###### *Antisocial Behavior*

Several studies tested the relationship between severity of pre-treatment externalizing/delinquent and aggressive behaviors and alliance quality, often with non-significant results (Alkalay, 2006; Cosgrove, 2020; Ryan et al., 2013; The Multisite Violence Prevention Project [MVPP], 2014) particularly when using non-shared measurement sources (Mattos et al., 2017). However, Ryan et al. (2013) demonstrated the importance of considering cultural factors as potential moderators. They found that for families identifying as Hispanic/Latino ethnicity, higher levels of externalizing behavior predicted lower caregiver-therapist emotional bonding. In contrast, for families identifying as African American ethnicity, higher levels of externalizing behavior predicted higher youth-therapist emotional bonding.

Other studies utilized official criminal history variables as potential determinants of alliance quality. In incarcerated males, Simpson et al. (2013) found that the number of prior convictions did not predict youth- or therapist-rated alliance overall but interacted with callous-unemotional (CU) traits to predict youth-rated alliance; at high levels of CU traits, more prior convictions predicted better alliances, but no significant relationship at low levels. In delinquent boys receiving residential treatment, Florsheim et al. (2000) found that more pre-treatment offense charges correlated with lower alliances across therapy, only for staff- not youth-rated alliance. In multivariate analyses, Ryan et al. (2013) found no evidence that pre-treatment arrests predicted youth- or caregiver-therapist bonding in multisystemic therapy (MST).

Prior/recent drug use was generally not associated with alliance quality, which held whether the alliance was rated by youth, staff, or caregivers (Alkalay, 2006; Florsheim et al., 2000; Ryan et al., 2013). However, further cultural comparisons by Ryan et al. (2013) suggested that higher youth-reported polysubstance use predicted lower ratings of caregiver-therapist bonding in Hispanic/Latino families but higher ratings of bonding in African American families.

###### *Mental Health and Personality*

Two studies evaluated the relationship between youth internalizing symptoms and the therapeutic alliance, with neither finding a significant relationship (Alkalay, 2006; Mattos et al., 2017). Bovard-Johns et al. (2015) similarly found that symptoms of trauma did not significantly predict alliance quality in young males adjudicated for sex offenses; an exception was post-traumatic sexual abuse symptoms, which predicted poorer alliances.

Two investigations evaluated the relationship between CU traits and the alliance, controlling for prior delinquency and treatment length. Mattos et al. (2017) found that higher levels of CU traits predicted positive youth-rated alliance three months after intake. Simpson et al. (2013) did not find a main effect of CU traits on youth- or therapist-rated alliance but observed an interaction between CU traits and the number of prior convictions (described above).

###### *Socio-Demographics*

There was little evidence for a direct link between youth socio-demographic characteristics (e.g., sex, age, race/ethnicity, economic status) and alliance ratings (Alkalay, 2006; Cosgrove, 2020; Hogue et al., 2006; Mattos et al., 2017; Ryan et al., 2013; Simpson et al., 2013; The MVPP, 2014). Regarding cultural background, for example, three independent studies showed that youth and parent alliance/bonding ratings did not significantly vary by self-reported African American, Hispanic/Latino, or Caucasian status (Alkalay, 2006; Hogue et al., 2006; Ryan et al., 2013; The MVPP, 2014). Conversely, one study found that youth who were Hispanic/Latino or Other race/ethnicity had higher alliances compared to Black/non-Hispanic youth, controlling for other covariates (Cosgrove, 2020).

###### *Family and Peer Factors*

Some studies investigated the relationship between family functioning/involvement and the alliance. The MVPP (2014) found that higher pre-treatment levels of parental involvement in a child’s education and parental monitoring predicted higher initial child-therapist alliance, whereas having an adult male in the home was linked to a higher parent alliance. Higher levels of parental monitoring also predicted shallower negative slopes (i.e., high initially that gradually declined) for child alliance throughout the intervention. In comparison, higher levels of parental discipline practices predicted steeper growth. A smaller-scale study found that youth classified into low- and high-alliance groups did not significantly differ on pre-treatment family cohesion and conflict (Alkalay, 2006).

Bovard-Johns et al. (2015) found that self-reported positive attachment to peers and communication with father independently predicted higher youth-rated alliance in males adjudicated for sex offenses. This is broadly consistent with the findings of Florsheim et al. (2000), who found that peer deviance at baseline negatively correlated with youth-rated alliance throughout treatment.

Some studies evaluated whether youth-therapist alliance quality was associated with parent-therapist alliance quality. Adolescent samples showed no significant correlations between alliance types (Hogue et al., 2006; Shelef et al., 2005) or small positive associations that increased moderately over time (Glebova et al., 2018; Ryan et al., 2013). In contrast, among younger children (6th graders), The MVPP (2014) found a strong positive relationship between initial child-therapist alliance and parent-therapist alliance.

###### *Treatment Perceptions and Expectations*

Several studies found positive moderate-to-large associations between alliance quality and youth level of treatment readiness, treatment fit with their theory of change (Savicki, 2008), and the perceived usefulness of treatment (Holmqvist et al., 2007). Similarly, The MVPP (2014) found significant positive correlations between initial parent and child satisfaction with the intervention and initial parent-therapist and child-therapist alliance, which covaried positively over time.

One study investigated youth and staff feelings toward one another and the relationship to their perceptions of alliance quality (Holmqvist et al., 2007). Although staff- and youth-rated feelings toward one another correlated with their respective perceptions of alliance quality, there was little significant correlation across measurement sources. An exception was that lower negative feelings toward staff rated by youth were significantly associated with higher staff-rated collaborative alliance.

###### *Therapist and Program Factors*

Few studies considered the impact of therapist characteristics on the alliance. Therapist experience (i.e., number of prior Functional Family Therapy [FFT] cases) was explored as a control variable in one study but did not significantly predict youth-rated alliance (Cosgrove, 2020). Two studies demonstrated positive associations between concurrent and cross-lagged measurements of caregiver-reported therapist adherence to the MST model and the alliance/bonding, particularly during the middle of treatment (Lange et al., 2017; Ryan et al., 2013). The link between therapist-youth (or family) race/ethnicity match and alliance quality was explored in two studies with neither finding a significant effect (Cosgrove, 2020; Ryan et al., 2013).

With respect to treatment type and focus, Hogue et al. (2006) found that observer ratings of the early adolescent-therapist alliance were significantly higher in the multidimensional family therapy (MDFT) group than in the individual CBT group. Drawing on the same sample, Dauber (2004) found that specific treatment focus areas within MDFT were not associated with alliance quality. Still, a CBT related treatment focus on peers related to higher alliance ratings.

##### Alliance–Outcome Relationship

Overall, 15 records provided quantitative data on the alliance–outcome relationship, synthesized below.

###### *Antisocial Outcomes*

Studies on the impact of the alliance on self- or informant-reported antisocial/delinquent and externalizing behaviors yield a complex picture of results. For example, some studies found no alliance–outcome effect (Alkalay, 2006; Dauber, 2004), positive effects for youths’ perspectives of alliance but not therapists’ (Diamond et al., 2006; Handwerk et al., 2008), or an effect for specific measures, time points, or subgroups of youth (Florsheim et al., 2000; Glebova et al., 2018; Hogue et al., 2006; Mattos et al., 2017; Shelef et al., 2005).

Still, other studies identify more nuanced associations between alliance and antisocial outcomes. Hogue et al. (2006) demonstrated that youth alliance did not predict declines in antisocial outcomes in individual CBT but did in family therapy, particularly for those where alliance improved during treatment. Florsheim et al. (2000) examined the effect of early and late alliance ratings on change in youth externalizing behaviors, controlling for several covariates. They found that early alliance ratings predicted increases in externalizing behaviors, whereas later ratings predicted decreases. This was partly explained by alliance change, where young people in a high stable/increasing alliance group were more likely to have reductions in staff-reported externalizing symptoms than those in a low stable/decreasing alliance group; this held only for staff-rated alliance and post-treatment outcomes, not follow-up outcomes. Cosgrove (2020) provides further support for the role of alliance change in reducing behavioral problems.

Evidence drawn from larger RCTs of adolescent substance use treatments indicates some positive effects of alliance on substance use outcomes. One study found higher parent-therapist alliance early in treatment predicted greater reductions in adolescents’ post-treatment drug use, but only among a high-treatment dose subsample (Dauber, 2004; Hogue et al., 2006). Other work shows positive effects of early youth-therapist alliance on follow-up substance use behaviors/symptoms, although effects diminished beyond six months follow-up (Diamond et al., 2006; Shelef et al., 2005). Expanding on these findings, Shelef et al. (2005) found a near significant youth X parent alliance interaction for post-treatment substance abuse symptoms. This suggested the positive effects of youth alliance were observed only when parent alliance was high or moderate, but not when low. The potential role of a strong parent-therapist alliance in reducing youth antisocial outcomes was highlighted in other work (Glebova et al., 2018), including a rigorous analysis that accounted for pre-treatment functioning, strength of the youth-therapist alliance, and utilized different measurement sources (Hogue et al., 2006).

Studies using official indicators of antisocial behavior indicated that neither youth nor therapist-rated alliance bore a relationship with residential incidents, violent institutional incidents, or prison rule violations (Handwerk et al., 2008; Savicki, 2008; Simpson et al., 2013). Evidence for the impact of alliance on recidivism was also limited, though some positive findings emerged. Holmqvist et al. (2007) found no significant correlations between youth/staff-rated alliance scores or clients’ conception of emotional bond reported during an interview, with post-treatment police reports or adjudicated sentences. However, higher staff-reported feelings of ‘closeness’ toward clients were associated with worse criminality outcomes. There was also a significant relationship between client conceptions of the usefulness of treatment at one-year post-discharge and reduced police reports, which the authors interpreted as supporting the importance of the alliance’s collaborative aspects (tasks/goals). However, clients’ outcomes might have biased their recollections of treatment usefulness.

In a sample of FFT completers, Cosgrove (2020) found that alliance and therapist fidelity worked in concert to protect against 12-month recidivism. Whereas alliance predicted fewer (any) arrests when controlling for a range of covariates, greater fidelity predicted reduced arrests that were adjudicated or convicted. Finally, Florsheim et al. (2000) found that youth with high stable/increasing alliances had significantly lower recidivism rates than those with low stable/decreasing alliances.

###### *Mental Health*

Three studies included internalizing symptoms as treatment outcomes, generally with no, small, or paradoxical alliance effects (Alkalay, 2006; Dauber, 2004; Handwerk et al., 2008; Hogue et al., 2006). One study reported a reduction in internalizing symptoms among youth with consistently high or increasing alliances over time (Florsheim et al., 2000).

###### *Parental Functioning*

Two investigations of family interventions evaluated the impact of alliance on parenting factors. The MVPP (2014) found that although the initial parent–provider alliance was negatively associated with a change in discipline practices post-treatment, the initial child–provider alliance positively affected outcomes. The authors queried whether restricted range in alliance scores might have contributed to the paradoxical effects, given the intervention’s highly structured, manualized nature. Furthermore, Glebova et al. (2018) found that caregiver-reported bonding with the therapist at the end of treatment was associated with positive caregiver perceptions of parental monitoring post-treatment; this did not generalize across rater perspectives.

###### *Treatment Progress, Attendance/Completion, and Fidelity*

Two studies found positive associations between bonding/alliance and staff assessments of goal attainment and program progress. However, in one instance, this held for therapist-rated but not caregiver- or youth-rated bonding (Glebova et al., 2018) and in the other, for concurrent but not prospective measurements (Savicki, 2008). Another focus has been on treatment attendance, with several studies providing limited support for an alliance association (Diamond et al., 2006; Hogue et al., 2006; Mattos et al., 2017; Simpson et al., 2013; The MVPP, 2014). An exception is Shelef et al. (2005), who analyzed a smaller sample from Diamond et al. and found higher parent alliance was associated with a higher likelihood of attending greater than 50% of sessions in MDFT. However, it is unclear whether this effect would persist in the presence of other covariates included in Diamond et al.

Regarding treatment completion, simple bivariate analyses suggest that lower youth alliance ratings may be linked with increased dropout (Florsheim et al., 2000), but multivariate analyses find no alliance main effect (Cosgrove, 2020). Interestingly, Cosgrove found that an increase in youth-therapist alliance relative to caregiver-therapist alliance predicted progress through FFT stages. This was suggested to reflect the alliance becoming more balanced between the family members, as caregivers had higher initial alliance ratings than youth.

Finally, three investigations evaluated whether the alliance influenced therapist adherence/fidelity. One study found no alliance effect in a highly structured, manualized intervention targeting violent sixth graders (The MVPP, 2014). In contrast, two studies describing home-based family interventions (MST and FFT) found positive cross-lagged associations between alliance and therapist adherence (Lange et al., 2017) and between alliance change and fidelity (Cosgrove, 2020). Lange et al. found these links were stable across youth characteristics, including age, gender, nonwestern ethnic origin, single-parent household, type/severity of problem behavior, and whether treatment was court ordered.

## Discussion

This review provides the first systematic synthesis of research concerning the therapeutic alliance in treating justice-involved and offending youth. We identified 23 independent studies meeting inclusion criteria, most of which were quantitative studies (61%) conducted in the US (74%). Males and racial/ethnic minority groups were over-represented, consistent with their disproportionate contact with juvenile justice systems. The synthesis of nine qualitative studies generated meaningful themes related to the development of a constructive alliance and the potential role of the alliance in initiating treatment change. The perspectives of young people were included in two-thirds of qualitative studies, with a small number drawing on therapist and parent views. The synthesis of 14 quantitative studies of determinants of alliance quality and its association with outcomes yielded mixed findings overall. Nevertheless, drawing from both syntheses, the review highlights several pertinent findings.

### Factors Linked to Alliance Formation

Contrasting with prior work that notes externalizing problems may hinder the alliance (Shirk & Karver, 2003), we found limited evidence to suggest antisocial problem severity predicted alliance difficulties in multivariate analyses. This discrepancy likely reflects differences in the reference group. In prior work, externalizing children are typically compared to internalizing children (e.g., Ayotte et al., 2015) whereas in this review, we focus only on offending/justice-involved youth. Interestingly, there was some evidence for a positive link between antisocial behaviors and youth/parent alliance quality, including among youth higher in CU traits. While some suggest this could indicate superficial alliances among high CU-youth (Simpson et al., 2013), others suggest it may reflect their increased interpersonal proficiency, verbal abilities, and emotion regulation skills (i.e., fewer barriers to alliance formation; Mattos et al., 2017). The review also draws attention to potential differences in the impact of problem severity on family-therapist emotional bonding across race and ethnicity (Ryan et al., 2013).

Consistent with theory on alliance formation (Orsi et al., 2010), the review highlights the potential relevance of the young person’s relational frameworks and social systems to therapeutic relationships. Positive peer and parent attachments were linked to positive youth perceptions of alliance quality (Bovard-Johns et al., 2015), and deviant peer relationships were associated with negative perceptions (Florsheim et al., 2000). Further, parents’ involvement in the young person’s life (e.g., in school, monitoring, limit setting) and therapy seemed to relate to better early alliances (Hogue et al., 2006; The MVPP, 2014), as did including a treatment focus on peers (Dauber, 2004). Thus, justice-involved youth with poor parental attachments, who are deeply embedded in deviant peer groups, and with family-related criminogenic needs, may be among the more resistant to treatment and the development of an alliance. These findings broadly align with the evidence on the effectiveness of multi-systems approaches for antisocial youth (Pappas & Dent, 2021) and extend this to suggest potential positive flow-on effects of these approaches for alliance formation. No studies examined the impact of youth attachment/interpersonal styles on the therapeutic bond, despite the disproportionately high rates of attachment-related abuse/neglect and interpersonal difficulties in this population (Modrowski et al., 2021). One study found that higher sexual abuse trauma symptoms predicted poorer alliances (Bovard-Johns et al., 2015), potentially via mechanisms of diminished trust and safety in relationships. The synthesis of qualitative studies highlighted the importance of attending to trust, safety, and security to facilitate an affective bond.

If young people (and their families) do not believe the treatment will benefit them, they are unlikely to enter a treatment relationship with enthusiasm (Kozar, 2010). It is unsurprising then that the review found youth with low readiness to change and negative perceptions of the utility of treatment and its “fit” with their ideas about change generally reported poorer alliances (Holmqvist et al., 2007; Savicki, 2008; The MVPP, 2014). This is consistent with research with justice-involved adults, where readiness is shown to be one of the strongest predictors of alliance (Taft et al., 2004). One interpretation is that alliance difficulties may partly mediate the link between lower treatment readiness/motivation and poorer treatment outcomes (Higley et al., 2019). Another perspective is that stronger alliances may improve young people’s perceptions of the value of treatment, their capacity to benefit, and their intrinsic motivation. A related theme arising from the syntheses was the importance of young people perceiving treatment goals and therapeutic tasks as helpful. Central to this was the collaborative and practically oriented aspects of the therapeutic relationship, where adolescents (and families) held more positive views of therapists who included their ideas about what they felt needed to change in goal setting, who helped them practically, and who maintained a focus on the ‘work’ of treatment (Holmqvist et al., 2007; see also Theme ii of qualitative results synthesis).

Despite the lack of attention paid to the role of therapist characteristics in quantitative studies, several qualitative studies identified the importance of therapists’ traits and skills in promoting positive alliances (see Theme ii). For example, qualities like empathy, genuineness, honesty, humor, flexibility/responsiveness, being ‘straight up’ (direct) with them, and a non-blaming, strengths-reinforcing approach were generally valued by adolescents.

### Alliance–Outcome Relationship

There was considerable variation in the impact of alliance on treatment outcomes for justice-involved youth. Both substantive (e.g., treatment modality and dose, types of outcomes, alliance type) and methodological characteristics (e.g., source of alliance and outcome assessment, timing of measures) appeared relevant to such variation. Most studies focused on antisocial outcomes (e.g., externalizing behaviors, substance use, institutional misconduct, recidivism), where most alliance effects emerged. More rigorous research is needed to understand the relations between the alliance and other clinical outcomes (e.g., mental health symptoms, parent functioning) and treatment processes (e.g., attendance, drop out).

A common finding was that a stronger youth-therapist alliance early in treatment rarely predicted post-treatment declines in externalizing behavior and offending and at times predicted worse outcomes. Evidence suggested this may be explained by how the alliance evolves: youth who began with poorer alliances that improved during treatment (or had high stable alliances) showed reduced antisocial outcomes, whereas deteriorating (or low stable) alliances were linked to problem escalation (Florsheim et al., 2000; Hogue et al., 2006). Thus, youth who enter treatment with low readiness/motivation (and hence poorer alliances) who are supported to become more collaborative and work-ready may achieve comparable outcomes to youth who maintain consistently strong alliances (Hogue et al., 2006). Conversely, alliance decliners, whether youth who experience an alliance rupture that is unrepaired or those who impression-manage early but become less inclined or able to sustain positive alliances as treatment demands increase, may be at risk of treatment failure (Florsheim et al., 2000). Although the potential impact of alliance change is clinically intuitive and broadly consistent with prior work (Polaschek & Ross, 2010; Shirk & Karver, 2003; Welmers-van de Poll et al., 2018), alliance shifts may be consequences rather than causes of therapeutic progress.

Only three studies examined specific features of the alliance in relation to outcome, with mixed findings. Simpson et al. (2013) found no alliance effect (overall or for tasks, goals, and bond components) on violent institutional incidents. Holmqvist et al. (2007) found staff ‘close feelings’ toward youth (i.e., affective relationship elements) were linked to worse offending outcomes. In contrast, youth perceptions of the treatment as useful (i.e., collaborative relationship elements) were associated with reduced offending. Another study found that the strength of the youth-therapist bond was not significantly related to any measured treatment outcomes (Glebova et al., 2018). Although these findings might suggest differences in the relative importance of the collaborative and affective aspects of the youth-therapist alliance in generating change for justice-involved youth, the evidence base is too small and limited to draw firm conclusions. In the broader youth therapy literature, meta-analyses find that the alliance-outcome effect is not significantly moderated by alliance dimension (McLeod, 2011; Welmers-van de Poll et al., 2018).

Family involvement and caregivers’ perceptions of alliance quality appeared relevant to the alliance–outcome relationship for justice-involved youth. Some work found that early/mid-treatment parent-therapist alliance better predicted improved antisocial outcomes than youth-therapist alliance (Glebova et al., 2018; Hogue et al., 2006). Other findings suggested the effect of youth alliance on antisocial outcomes may be enhanced in family-based treatment and when a strong parent alliance is developed (Hogue et al., 2006; Shelef et al., 2005). The risk of premature dropout may also be reduced where youth and parent alliances become increasingly balanced over time (Cosgrove, 2020). The parent alliance–outcome relationship has been demonstrated in the wider youth therapy literature (Karver et al., 2018; McLeod, 2011). However, it may have particular importance in youth justice settings, where a parents' lack of involvement and negative beliefs about treatment are among the most common barriers to service provision for justice-involved youth (Kapoor et al., 2018). Although there have been increased efforts to involve parents/caregivers, many youth justice and forensic mental health services lack resources or frameworks to actively engage families in treatment (Robertson et al., 2019). Therefore, while the review findings require replication, they suggest increased research and practice focused on the role of parent alliance in treating justice-involved youth may be worthwhile, including how therapists and organizations can best facilitate these alliances.

Few quantitative studies compared the relative contribution of treatments’ technical and relationship aspects to outcomes with justice-involved youth. One exception showed that both were important in predicting outcomes following FFT, with alliance being more salient for clinical externalizing symptoms and fidelity for program retention and adjudicated arrests (Cosgrove, 2020). Although these findings reflect a single study, the synthesis of qualitative studies also supported the complementary role of treatment techniques and alliance in initiating and sustaining change (i.e., Theme iii). The bidirectional link between alliance and therapist fidelity found in this review further underscores the need to include technical and relational process measures to understand better how they interrelate and their unique and interactive effects on outcomes in this population.

### Limitations and Future Directions

There are several limitations associated with our review. First, the number of included studies was small, and the evidence that emerged was often inconsistent. A greater volume of research is needed, which would be assisted by therapists and program evaluators including measures of the alliance in treatments with justice-involved youth. Second, the quality appraisal of studies identified several issues, particularly problems with sample representativeness (e.g., exclusion of treatment dropouts), confounding influences, limited consideration of whether a change in alliance reliably relates to a change in outcome, and a lack of detail about treatment fidelity. These issues could in part be addressed by researchers striving for random sampling, considering key confounds of their hypothesized relationships and controlling these through design/analysis, employing longitudinal designs with repeated measurements of both alliance and outcomes, and sufficiently detailing methods and treatment protocols. Third, alliance measures used were diverse (nine different measures across 14 quantitative studies) and not always widely validated. This diversity mirrors the variation in how researchers define youth alliance (Karver et al., 2018), which likely contributed to the inconsistency of findings. More empirical and conceptual work is required to examine which measures and what features optimally capture the therapeutic alliance in youth justice settings. While this work progresses, researchers’ selection of alliance measures should ideally be guided by theory, how reliable and valid the tools are with youth (offending) populations, and prior research demonstrating their sensitivity to change. Assessment from multiple perspectives is also recommended. Overall, these methodological issues mean the review findings should be viewed as important preliminary data about the alliance in justice-involved youth that must be re-evaluated as more high-quality evidence emerges.

The review also highlighted some important knowledge gaps and avenues for future research, several of which have already been discussed. Regarding determinants of alliance quality, more research is needed examining the role of therapist characteristics and in-treatment behaviors, parent characteristics, and other treatment setting/contextual factors (e.g., group vs. individual therapy, telehealth vs. in-person interventions, level of actual or perceived coercion, institutional climate and safety, quality of relationships with other involved therapeutic or support staff). Justice-involved youth are likely to come from diverse cultural backgrounds, and so greater attention should be given to understanding whether and how culture, language, and perceptions of cultural safety affect the therapeutic alliance. Also lacking from studies was in-depth information from therapists' perspectives about how they form productive alliances with diverse clients and their families. Increased research focus in these areas would advance theory and knowledge and provide practical information for clinicians about strategies to build the alliance. Finally, more research is needed examining the mechanisms through which the alliance might work to influence outcomes, the features (e.g., affective versus collaborative) that may be most relevant to treatment change, and the conditions impacting whether this occurs.

## Conclusion

This review draws three overarching conclusions about the therapeutic alliance in treating offending/justice-involved youth. First, there is insufficient evidence to conclude that alliance quality consistently and causally predicts significant reductions in antisocial behavior. Whether the inconsistency reflects unique complexities of the alliance–outcome relationship in the youth justice context or is driven by the small and methodologically varied evidence base is unclear. Nevertheless, some promising preliminary findings about the alliance–outcome relationship emerged. Most notably, treatment benefits are likely associated with alliance growth and creating positive alliances with caregivers. Second, while the determinants of alliance quality remain vague and under-researched, the review highlights the potential relevance of the young person’s relationships with peers and parents, and their treatment readiness and expectations, to the quality of their alliance. Third, the thematic synthesis of qualitative research supports the importance of creating an environment of self-determination and collaboration, drawing on personal strengths, and active therapeutic engagement in building the alliance, along with the role of therapist qualities and attending to issues of trust, respect, and safety in forming positive bonds. Despite the equivocal findings on the alliance-outcome relationship from quantitative research, the qualitative synthesis suggests that building positive alliances may establish a foundation for initiating change. A greater volume of research is needed to overcome some of the limitations identified to understand the alliance's role in treating justice-involved youth more precisely.

## Data Availability

This is a systematic review, which relied upon existing data that are publicly disseminated. No primary data were generated, and we did not conduct meta-analyses.

## Footnotes

1 The working alliance is a broader alliance conceptualization that typically includes applications outside therapeutic settings.
