# Correction: Determinants and Outcomes of the Therapeutic Alliance in Treating Justice-Involved Youth: A Systematic Review of Quantitative and Qualitative Research

**DOI:** 10.1007/s10567-022-00410-7

**Published:** 2022-09-08

**Authors:** Nina Papalia, Ashley Dunne, Natasha Maharaj, Erika Fortunato, Stefan Luebbers, James R. P. Ogloff

**Affiliations:** 1 Centre for Forensic Behavioural Science, Swinburne University of Technology and Victorian Institute of Forensic Mental Health, Level 1, 582 Heidelberg Road, Alphington, VIC 3078 Australia; 2 Psychology Department, John Jay College of Criminal Justice, New York City, NY USA; 3 Youth Forensic Specialist Service, Alfred Health, Moorabbin, VIC Australia

## Correction to: Clinical Child and Family Psychology Review 10.1007/s10567-022-00407-2

In the original published article, an error was introduced in a table note under Table 1 and Table 2 and has since been corrected. The table note for Table 1 and Table 2 in the original published article read, “Bold rows reflect studies with overlapping samples”, but should have read, “Adjacent bold rows reflect studies with overlapping samples.” That is, study samples from Dauber (2004), Alkalay (2006) and Hogue et al. (2006) are overlapping, and study samples from Shelef et al. (2005) and Diamond et al. (2006) are overlapping. The original article has been corrected.

